# Sustainable financing for municipal solid waste management in Nepal

**DOI:** 10.1371/journal.pone.0231933

**Published:** 2020-08-20

**Authors:** Bishal Bharadwaj, Rajesh Kumar Rai, Mani Nepal

**Affiliations:** 1 School of Earth and Environmental Science, The University of Queensland, Brisbane, Australia; 2 Ministry of Federal Affairs and General Administration, The Government of Nepal, Singadarbar, Kathmandu, Nepal; 3 The Center for People and Forests (RECOFTC), Bangkok, Thailand; 4 South Asian Network for Development and Environmental Economics (SANDEE), International Centre for Integrated Mountain Development (ICIMOD), Kathmandu, Nepal; Kyonggi University, REPUBLIC OF KOREA

## Abstract

Financing municipal solid waste (MSW) services is one of the key challenges faced by cities in developing countries. This study used plastic waste, a constituent of MSW, to explore the possibility of generating revenue for financing MSW management in the municipalities of Nepal. The results of this study suggest that plastic material recovery could generate revenue, which is equivalent to 1.38 times of the plastic-waste-related management cost when collection efficiency reaches 66.7%. An increase in 1% of recovery rate and collection efficiency could cover an additional 4.64% and 2.06% of the costs of managing plastic waste, respectively. In addition, an increase in tax on imported plastic materials could also motivate recovery of plastic waste for recycle and reuse. An additional 1% tax on plastic imports would be sufficient to cover plastic-related waste management when plastic waste recovery and collection efficiency rates are low. This plastic recovery- revenue exercise could be expanded to other materials such as paper and metal to fully understand the possibility of sustainable financing of MSW management and reducing environmental harm in developing countries like Nepal.

## Background

There has been a steady increase in the urban population worldwide over the years. According to a report published by the Department of Economic and Social Affairs of the United Nations, the world’s urban population was 55% of the total population in 2018 but is expected to increase up to 68% by 2050 [1]. According to the Report, more than 90% of this growth would take place in Asia and Africa. The increase in urban population, coupled with economic growth and improved living standards, has resulted in the generation of enormous amounts of waste already in cities in developing countries [2]. But municipal solid waste (MSW), if not managed properly, produces negative externalities and contributes to flooding and waterlogging during extreme climatic events such as excessive rainfall [3–6].

Municipal authorities are working to make their cities resilient and smart. Smartness aims to improve the welfare of citizens by making cities liveable [7]. Although MSW management is one of the major components of making cities resilient and smart [8,9], it remains a major challenge for municipal authorities, particularly in developing countries [10]. Unplanned settlements, poor infrastructure, inadequate resources and capacities, and low level of awareness among municipal residents are making MSW management all the more complex [11,12]. For many municipal authorities in developing countries, solid waste management is a high cost activity, which may command up to 50% of the total municipal budget [13,14].

Additional infrastructure, mainly physical, to manage MSW may not seem an attractive option to municipal authorities under severe resource constraints. But there is a growing demand for better solid waste management services by residents who are also ready to pay increased waste collection tariffs for improved services [9,15]. Compounding the problems are the shortening life-spans of landfill sites due to the percentage increase in plastic waste which takes a longer period of time to decay [16]. The demand for more landfill sites is expected to increase in future because of the growing consumption of processed food products that are packaged in plastic (among them, bottles, food wraps, bags, etc.) and the use of electronic appliances which take a long time to decompose. In the absence of a mechanism for proper recycling of plastic and electronic waste (e-waste), the demand for landfills sites is bound to increase steadily, which would only add to conflicts between municipal authorities and communities close to the landfills, as landfill sites generate disamenities to nearby residents [17].

Dumping of plastic waste in rivers and canals, in addition to drainage systems, results in flooding and water logging in low-lying areas. In such situations, structural interventions can provide only a short-term solution in the absence of MSW management [6]. Similarly, imposing a ban on plastic use without strict enforcement may not work properly [18]. Hence, reduction of plastic waste at source is critical for preventing water logging and flooding in cities and low-lying areas. Several cities have, in fact, enforced a levy on the use of plastic bags to reduce its release to the environment [1] and studies show that a levy on disposable plastic bags reduces its use [2]. Some European countries have introduced policies on recyclable packaging standards to increase recyclable waste [4]. While the management of electronic waste is challenging, discussions already are underway in developed countries to implement extended producer responsibilities [5].

In this context, the present study attempts to answer how to make MSW cost-effective and financially sustainable. Although many studies have explored the financial contribution of households, businesses and institutions to MSW management [9,19], they have not been of much use to policy makers and municipal authorities in developing countries who seek information on a national-scale sustainable financing mechanism for managing MSW. Sustainable financing, as understood in the present case, is one where MSW management activities would not require additional funding from other sources but one where material recovery and recycling alone would generate sufficient resources.

This study examines both sides of the financing mechanism–costs and revenues–for properly managing plastic waste. It also estimates the additional tax that needs to be imposed at national level on the import of plastic materials when material recovery and collection efficiency rates are low. This study hypothesizes that the additional revenue could be used for managing plastic waste so that municipalities would not need to overly rely on the voluntary subscriptions of households to carry out solid waste management services. The current practice in Nepal is one where households have the option of subscribing to the MSW collection service by paying a pre-specified tariff. In comparison, the proposed imposition of additional tariffs on the import of plastic materials or the recovery of plastic waste for recycling would be more inclusive while ensuring distributional justice vis-à-vis the poor and vulnerable groups living in urban slums and other areas, who are unable to afford the service charge in the existing pay-as-you-use waste management service.

## Methods

### Theoretical framework

Since MSW management is a complex process involving a series of steps with multifaceted effects on human life, its improvement requires the participation of diverse stakeholders and treatment of waste as a resource while taking into consideration cross-cutting issues like sustainability, inclusion, gender and governance. This can be achieved through a careful study of the different components and contextualization of standard practices.

An integrated and sustainable solid waste management (ISSWM) approach views solid waste management as a system which engages all stakeholders by adopting the principles of equity, effectiveness, efficiency and sustainability. This approach basically considers three factors–(i) stakeholders, (ii) elements, and (iii) aspects–in order to design the MSW management system focusing on reduce, reuse and recycle [20]. This concept, often called the 3Rs, is predicated upon the belief that solid waste comprises several types of materials with different values and impacts. The analysis in this study mainly focuses on plastic waste, which is considered one of the most challenging categories of waste [18].

As most of the plastic waste could be recycled, the ISSWM promotes a circular economy which would be able to generate enough resources to pay off the management costs [21]. But, in order to be feasible economically and technically, MSW management should factor in social acceptance, cost effectiveness and technical feasibility.

**Fig 1:**
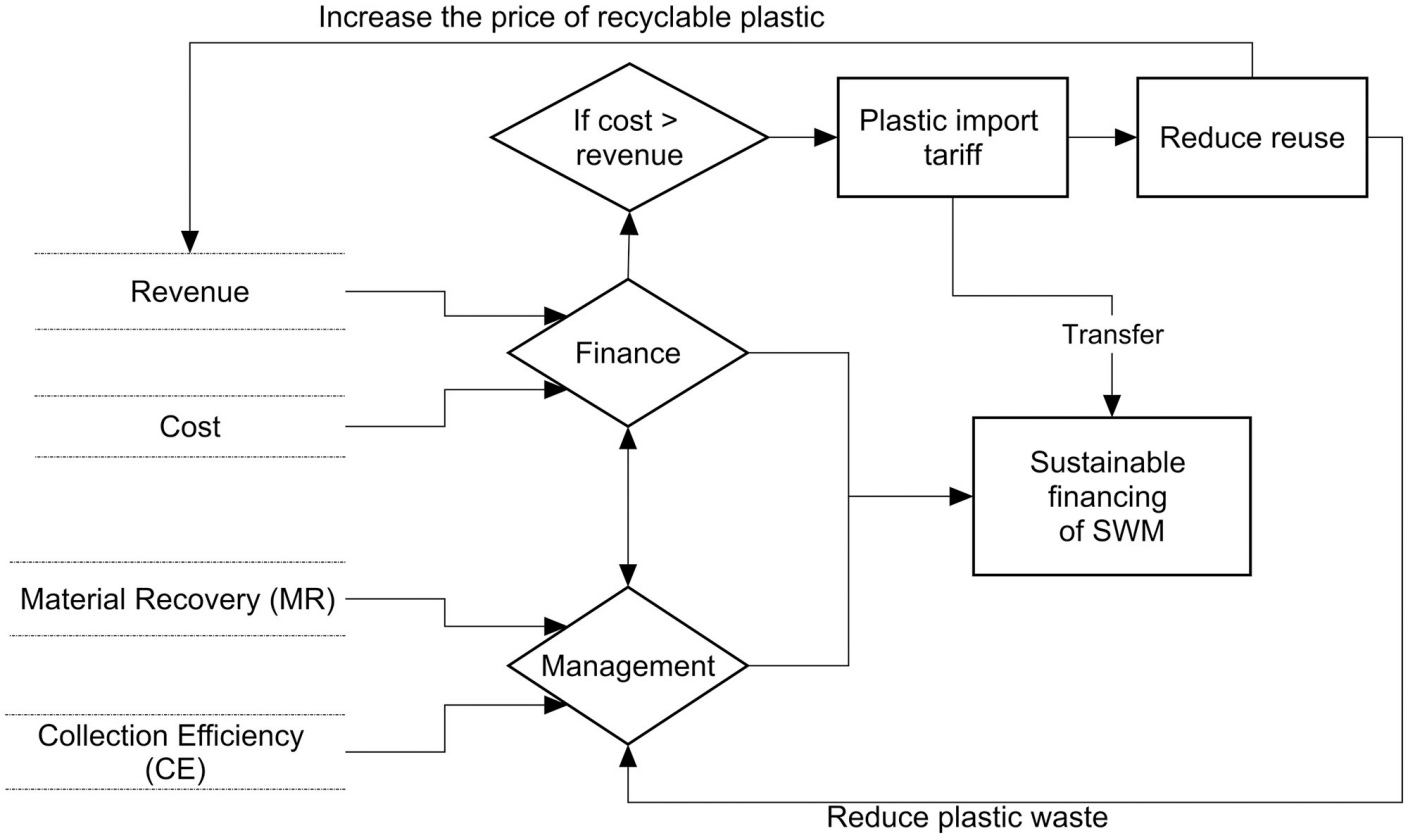


Fig 1 depicts the framework for sustainable financing of MSW management services. The framework indicates that sustainable financing of MSW management has two components: (i) financing and (ii) management of waste. Financing depends on costs of management and revenues from recycling and reusing. Management costs can be minimized by reducing the use of plastics, increasing the reuse of waste materials, and generating lower volumes of waste.

Similarly, management of wast has two aspects: material recovery and collection efficiency. Material recovery is the percentage of total recyclable material recovered from collected solid waste. Collection efficiency which is the percentage of recyclable waste material collected out of the total waste, influences the cost of MSW management while revenue relies on material recovery, collection efficiency, and price of recovered materials. In Nepal, very few households practice separation at source; even if households segregate waste at source, service providers generally do not have a separate pick-up service [9]. Some households practice separation of saleable materials such as metal, plastic and paper from the waste. Generally, materials that are recovered for reuse or for sale by households are not included in total waste estimates in this study. Hence, this study only considers recovered items from the landfill sites.

The financial component (revenue and cost) is market driven. Therefore, in this analysis, revenue and costs are taken from the existing waste management practices in Nepal. As for the management component, government policies in addition to efforts of municipal authorities could improve it. For instance, the quantity of recycled materials depends on the quality of plastic materials used in packaging [22], which highlights the role that the government can play in imposing restrictions on the quality of plastic used in packaging. Similarly, provision of incentives to households to segregate waste at household level would contribute to improving collection efficiency.

The deficit in the cost of plastic waste management could then be made up by imposing an additional tariff on the import of plastic materials that are mainly disposable and thus end up in landfills and drainage systems. The additional tariff would make the use of such plastics relatively expensive, which would help thereby to: a) promote substitutes, b) reduce the use of plastics, and c) increase the price of recycled plastic, which would increase revenues. Since plastic waste will become expensive with the additional tariff, it would help promote recycling as well as reduce the use of plastics.

### Data collection

This study used both qualitative and quantitative approaches to collect data. A total of 83 respondents from different stakeholder groups were interviewed to explore existing MSW management and financing. Of them, 20 were collectors of recyclable materials, 23 were local government officials, 15 were environmental activists, and 25 were policy makers. These respondents were chosen using different approaches, based on the category. For example, the collectors of recyclables were surveyed from the selected municipalities. These collectors generally gather in local tea-shops in the morning for tea. The research enumerators approached them and obtained their consent to answer the relevant questions. This process was continued in the different municipalities until 20 respondents were interviewed. Other categories of respondents, such as local government officials, environmental activists and policy makers, were easier to identify. We prepared the list of potential respondents and discussed with them first their availability for an interview as well as interest in providing information voluntarily.

The questionnaire, which is in two parts, has been submitted to the journal for public access. The first part aimed at collecting data on existing MSW management, which was the same for all respondents. The second part sought to collect information on the involvement of particular stakeholders in MSW management and the supply chain of recovered material. The information provided by private companies that collect and dispose of MSW was used to determine the material recovery rate (MRR).

So far, there is no institutional review board or national ethical guidelines for social science research in Nepal. Therefore, the study adhered to established standards in research ethics such as obtaining verbal consent for participation in research, keeping personal informal confidential, and allowing participants to quit the discussion at any point withdrawing their consent if they so desired. Furthermore, no personal information of the key informants or other individuals, who provided information related to solid waste generation and import of plastics or related information, were used in this research. Average values of the collected information were used for developing different scenarios for simulation.

For this research, the material recovery rate is estimated using a series of Eqs (1)–(5) based on the SWM baseline data of municipalities taken from the report published by the Asian Development Bank (ADB) in 2013 to assess the current situation of SWM in municipalities of Nepal [23]. The Tables in Appendix 4–7 were used to estimate the efficiency of waste collection, weight of the solid waste estimates in municipalities, and the contribution of plastic waste in the total waste. The Table in Appendix 9 of the ADB 2013 Report was used to calculate the expenditure incurred by a municipality to manage a ton of solid waste. Missing data were excluded while calculating the average value of the relevant variables across the municipalities. The data collection process with the major findings of the baseline survey can be accessed from the Asian Development Bank’s website [3].

CW=TW×ACE (1)

where,

CW is weight of waste collected by municipality

TW is total weight of solid waste produced in the municipality

ACE is average collection efficiency

MRP=RPM×CW (2)

where,

MRP is weight of the material recovery potential waste

RPM is percentage of recoverable potential material in CW

MR=MRP*MRR (3)

where,

Material Recovered (MR) = weight of material recovered (for this study plastic) from MRP

MRR is material recovery rate which is the percentage of particular material that can be recovered from the MRP.

PTWR=MR/TW (4)

where,

PTWR is proportion of recovered waste to TW

TMR=PTWR*PCW*UP (5)

where,

TMR is total material recovered

PCW is weight of waste produced per capita

UP is population living in municipalities

The amount of solid waste materials was estimated for commercial, institutional and residential waste separately as the material composition varies by source of waste [23]. MRR affects overall calculation of material recovered, and costs and revenue from recycling. Therefore, we cautiously selected the MRR. We first reviewed the material recovery rates for different countries and compared them with local rate. in different municipalities in Nepal. The material recovery rates in some of the municipalities in Nepal were similar to those observed in developed countries. An example would be Dhankuta Municipality, where solid waste management has been practiced for the past several years, the rate of which is comparable to that reported in Australia for 2012 [24].

The cost of and revenue from MSW management were estimated using material recovery information and the results were simulated using two additional items of information: a) average price of recyclable materials and b) MSW management cost. The potential revenue gap was then estimated, which was followed by the estimation of the levy to be imposed on imported plastics to cover the resource gap (cost–revenue).

Plastic import data for Nepal were obtained from the Department of Customs under ‘Plastic and article there-of’, which was classified under HS code 39 for the years 2010–2016. This information was used to analyse the import values of plastic-related materials and the taxes collected under different headings. The additional levy on plastic imports based on the resource gap was estimated thereafter. In doing so, we expect the top-up levy to increase the price of plastic materials reducing in turn the demand. The price mechanism would provide some incentive for using alternative materials in place of plastics while also reducing the demand for disposable plastics as the price goes up, thus curbing the increasing per capita consumption of disposable plastic items. Already, there have been instances reported in Nepal where large retail stores have stopped providing plastic bags to their customers while some restaurants have started using local materials such as bamboo utensils and dried-leaf plates as substitutes for plastics. With an additional import duty on plastic materials, the trend in using substitutes is bound to increase with time.

### Analysis

The analysis determines the different components of sustainable financing (see Table 1). It uses information for 58 municipalities as Nepal had only 58 municipalities till 2013 while the rest were village development committees [23]. After the promulgation of the new Constitution in 2015, there has been a drastic change in the number of local administrative units in Nepal with, currently, more than 750 local administrative units across the country. For our analysis, we considered the earlier administrative structure and have thus included 58 municipalities in the study.

**Table 1:** Levels of different components of sustainable financing.

Finance		Management	
Revenue	Cost (per ton of waste collected)	Material recovery	Collection efficiency
R1- NPR 30/kg R2- NPR 15/kg R3- NPR 12/kg	C1- NPR 2,347/ton C2- NPR 4,673/ton	Low -12% Medium -15% High—30%	Least efficient—20% Existing—33.7% Medium—50% High—66.7% Maximum—90%

NPR is Nepalese Rupees; USD1 = NPR 85 in 2012

In the analysis, two types of MSW management costs were identified: (i) lowest cost (C1) and (ii) average cost (C2). The lowest cost (C1) is the cost of MSW management practiced by Dhankuta Municipality, which has one of the best MSW management practices in Nepal. The average cost (C2) is the average cost of MSW management practiced by the other municipalities in Nepal. Similarly, three revenue scenarios were determined based on the price of recycled/reused plastic materials: (i) average price received by collectors (R1), (ii) price received by segregators in Dhankuta municipality (R2), and (iii) average price received by households (R3). The prices for R1, R2 and R3 were elicited, respectively, from the collectors’ survey, a manager of the solid waste management company contracted by Dhankuta Municipality, and the website of Khalisisi–a social enterprise engaged in door-to-door collection of recyclable material [25].

Based on the costs and revenues in Table 1, six scenarios were developed to assess the costs and benefits from solid waste management. These scenarios are R1C1, R1C2, R2C1, R2C2, R3C1 and R3C2. Of these scenarios, R1C1 is the best with the best selling price for recovered plastic materials with the least management cost. In contrast, R3C2 is the worst scenario with regard to the average MSW management cost; it also has the lowest price for recovered plastic.

These scenarios were assessed using different material recovery rates and collection efficiencies as reported in Table 1 (columns 3–4). The estimated collection efficiency for Nepal is 33.7%, which is the weighted average of collection efficiency of the 58 municipalities in 2012. As communicated by the environment officer of the municipality, the plastic recovery rate is between 12% and 15% in Dhankuta municipality minus the segregation of plastics and other waste at source. It is expected that segregation at source will increase the recovery rate up to 30% [26]. Based on these findings, three different scenarios of plastic recovery rates are identified: 12%, 15% and 30%. Similarly, collection efficiency is categorized into five groups: (i) Least efficient–below average, (ii) Existing–equivalent to the average of the disposed waste in landfills, (iii) Medium–the performance of smaller towns with better management, (iv) High–the lower bound of major cities, and (v) Maximum–the higher bound of major cities.

## Results

### Material recovery potential

In 2012, Nepal imported 0.4 million tons of plastic while the estimated plastic waste generated was 0.23 million tons [27]. On average, the 58 municipalities generally produced 1,281 tons of waste per day of which 769 tons were household waste, 447 tons commercial waste and 65 tons institutional waste [23]. Organic waste exceeded 66% of the total waste volume generated at the household level while it was 40% and 20%, respectively, in the case of commercial and institutional waste [23].

The average MSW collection efficiency of all municipalities is 33.7% in Nepal [28]. Based on the collection efficiency, the daily quantity of materials that can be processed for material recovery is 432 tons. The daily material recovery potential is thus 196 tons, which is around half of the collected waste. Organic material is recoverable or reducible and many households use organic waste as animal feed or, once processed, as compost for their farm or kitchen garden. However, organic waste in big cities is becoming unmanageable since, in big cities like Kathmandu, plot sizes are smaller, kitchen gardens are absent, and many families live in rented apartments, limiting the scope for composting the organic waste in their backyards.

Another recoverable material is paper. On average, 48.7 tons of paper can be recovered on a daily basis from these municipalities. There is a wide variation in the price and quality of paper. Khalisisi, an organization working on waste recycling in Nepal, pays NPR 17/kg for notebooks, NPR 9/kg for cardboard, NPR 11/kg for used books, and NPR 14/kg for newspapers [25]. As there is a chain of collectors, an increased volume of collection can be sold to big collectors which could bring in a better price for the sellers.

Plastic is a dominant material in waste. At present, on average, 6.05 tons of plastic waste can be recovered daily from the 50.4 tons of plastic waste produced each day. According to collectors, the rate of recovery varies with the type of the plastic product. For example, while the recovery rate of plastic bags is 58%, for bottles it is 61% and for utensils it is 62%. These recovery rates can go up higher when waste is managed more efficiently. But there are technological constraints to recycling since some plastic items are not recyclable while many items do not have enough volume. Low volume is not profitable as the marginal cost of recycling exceeds the marginal benefits from recycling.

**Fig 2:**
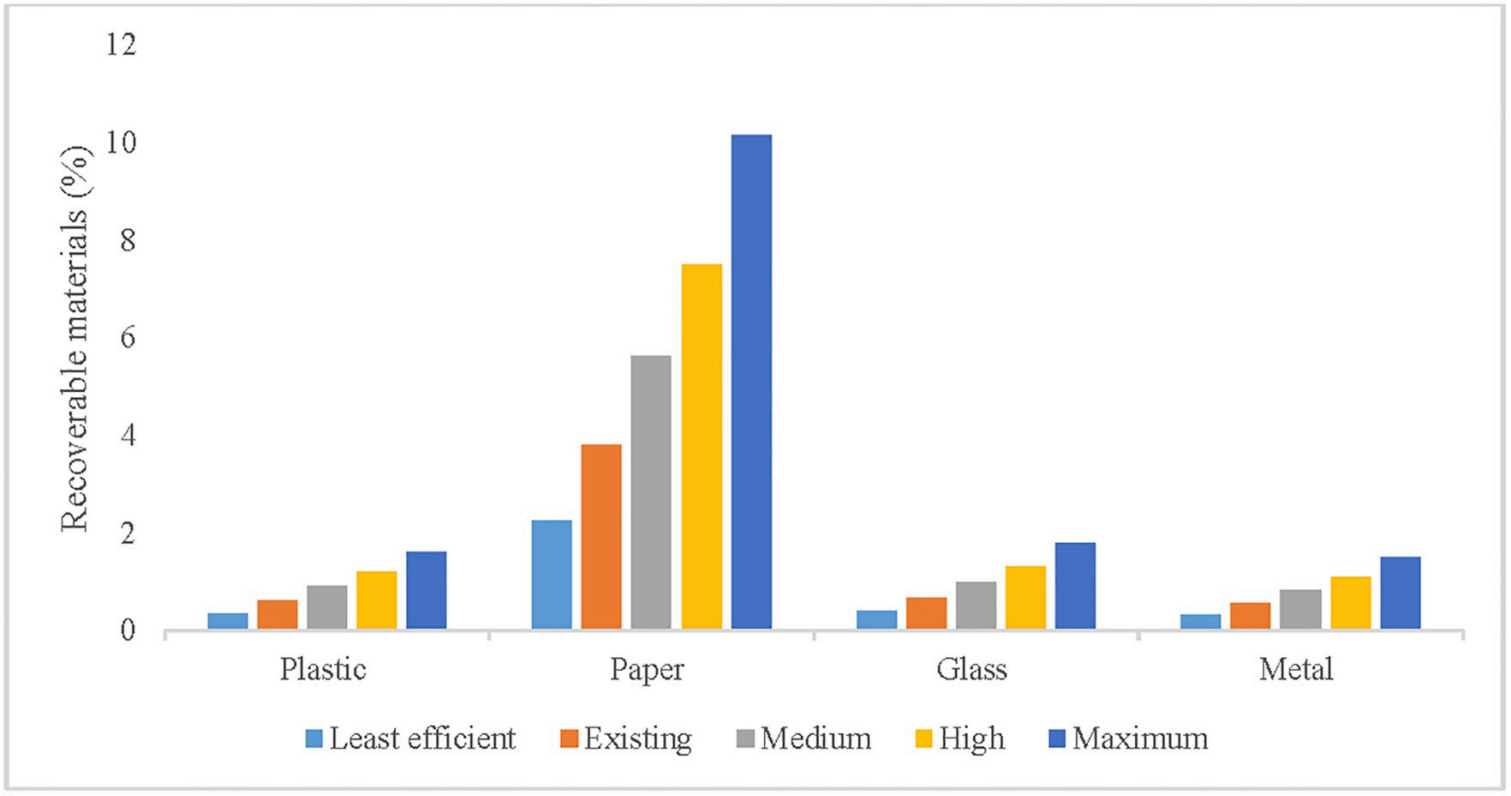


The quantity of recoverable materials changes with a change in collection efficiency (Fig 2). Based on the existing collection efficiency and the 12% plastic MRR, only 0.61% of the total waste produced can be recovered as plastic. The maximum plastic recovery would thus be 1.62% of the total waste produced at 90% collection efficiency with 12% MRR. Given this data, a 1% increase in plastic MRR would increase recovery by 0.04% of plastic waste and 0.02% of total solid waste.

### Financing

Table 2 reports the costs and revenues at 12% MRR in different collection efficiencies for the best and worst case scenarios. The results suggest that, at the existing collection efficiency, the revenue generated from recovered plastic could cover 1.6% and 7.8% of the total solid waste management costs, respectively, in the worst and best case scenarios. Similarly, it could cover 11.2% and 55.7% of the plastic-related MSW management costs in the worst and best case scenarios, respectively. As such, the management would require additional finances to cover the plastic-waste-related cost, which is equivalent to 0.2% and 0.7% of the total plastic import value, in the best and worst case scenarios, respectively.

**Table 2:** Cost and revenue in best case (R1C1) and worst case (R3C2) scenarios at 12% recovery rate.

Criteria	Collection efficiency									
	Least efficient (20%)		Existing (33.7%)		Medium (50%)		High (66.7%)		Maximum (90%)	
	R1C1	R3C2	R1C1	R3C2	R1C1	R3C2	R1C1	R3C2	R1C1	R3C2
% of overall MSW management cost	4.6	0.9	7.8	1.6	11.6	2.3	15.4	3.1	20.8	4.2
% of plastic-related MSW management cost	33.0	6.6	55.7	11.2	82.6	16.5	110.0	22.1	149.0	29.8
Revenue (NPR in million)	30.0	12.0	51.0	20.0	76.0	30.0	102.0	41.0	137.0	55.0
Top-up to cover plastic waste related cost (% of import value)	0.3	0.8	0.2	0.7	0.1	0.7	0.0	0.6	-0.2	0.6

Simulation results using primary and secondary sources of data discussed under data section.

The results indicate that if collection efficiency increases to 66.7%, then the revenue generated from the recovered plastic at 12% MRR would outweigh the plastic-related MSW management costs in the best case scenario while it would cover only 22% of the costs in the worst case scenario. Even if collection efficiency improves to 90% in the worst case scenario, the revenue from the recovered plastic would cover a mere 30% of the plastic-related MSW management cost.

### Sensitivity analysis

An optimistic scenario is defined as a case where the plastic MRR is 15% and the collection efficiency is high (at 66.7%). This scenario was set up after a series of discussions with the relevant stakeholders and officials of municipalities assuming that it would be achievable in the Nepalese context. The results of the optimistic scenario for best and worst cases are reported in Table 3. The results suggest that the best case scenario generates 1.38 times more revenue than the plastic-related MSW management cost earning NPR 35 million whereas the revenue generated from recovered plastic in the worst case scenario can cover only 27.6% of the plastic-related MSW management cost. The optimistic condition will prevent 4,220 tons of plastic from entering the environment annually while reducing 1.5% of total waste from the waste stream.

**Table 3:** Results of optimistic scenario.

Criteria	Unit	Best Case Scenario (R1C1)	Worst Case Scenario (R3C2)
**MSW management cost (per year)**			
Total cost (A)	NPR Million	659.0	1312.0
Plastic proportionate (B)	NPR Million	92.0	184.0
**Plastic revenue (C)**	NPR Million	127.0	51.0
Deficit (D) for managing plastic waste = [B-C]	NPR Million	-35.0	133.0
**Cost recovered from recovered plastic revenue**			
Total MSW management cost (C/A)	percentage	19.3	3.9
Plastic proportionate (C/B)	percentage	138.0	27.7
Top-up (D/total value of import)	ratio	-0.16	No top-up required
Weight of recovered plastic proportionate to total waste	percentage	1.50	

Simulation.

In addition, two analyses were carried out to examine how much the revenue from recovered plastic is sensitive to collection efficiency and recovery rate (see Table 4). The effect of recovery rate on plastic revenue is calculated at existing collection efficiency (33.7%) and the effects of collection efficiency on the 15% recovery rate. The results show that a 1% increase in recovery rate and a 1% increase in collection efficiency would recover an additional 4.64% and 2.06% of the plastic-related MSW management cost, respectively. Similarly, recycled plastic waste would increase by 142 and 63 tons, respectively, with a 1% increase in both recovery rate and collection efficiency.

**Table 4:** Plastic-related cost recovery for the best case scenario (R1C1).

Existing collection efficiency (33.7%)			15% recovery rate		
Recovery rate (%)	Cost recovery (%)	Plastic recycled (ton)	Collection efficiency (%)	Cost recovery (%)	Plastic recycled (ton)
12	55.7	1,707	20.0	41.3	1,266
13	60.3	1,849	33.7	69.6	2,134
14	65.0	1,991	50.0	103.3	3,165
15	69.6	2,134	66.7	137.7	4,220
30	139.2	4,267	90.0	185.9	5,698

Simulation.

The results also suggest that a 30% recovery rate generates 39% more revenue compared to the plastic-related MSW cost when recycling 4,267 tons of plastic per year. Similarly, revenue generated from recovered plastic at collection efficiency more than 50% can cover the plastic-related MSW management cost by reducing at least 3,165 tons of plastic in the waste stream. Table 4 suggests that with a 30% recovery rate under the existing collection efficiency rate, the plastic recycling program would generate enough revenue to finance the costs of plastic waste management. On the other hand, with a 15% recovery rate, a 50% collection efficiency would be needed to generate enough revenue to finance the plastic waste management costs.

### Discussion

The volume of MSW is showing an increasing trend in Nepal as the economy undergoes a transition from a farm-based rural economy to an industry- and service-based urban economy [29,30]. With this transition, consumption of processed foods packaged in plastic covers/containers has been on the rise. Furthermore, the use of plastic bags has been rampant as these bags are mainly provided at no additional cost at groceries and shopping centres. In urban Nepal, on average, a household uses over 10 plastic bags per week for groceries which translates into roughly 28,000 tons of plastic bags in the environment per year if not recycled properly [18]. For this estimate, a conservative weight of 1 plastic bag equals 10 grams had been used whereas a plastic bag used in homes could weighbetween 8 and 32 grams.

The local municipal authorities have been facing challenges in managing solid waste effectively because of poor planning, a low level of awareness among residents, and lack of resources. But the increased volume of waste is likely to increase the cost of MSW management. Household waste collection is one of the most expensive MSW management activities in Nepal. Sweeping and waste collection comprise around 60–70% of the total MSW management cost of municipalities [23]. In addition, a challenge arises relating to the management of land-fill sites with increasing waste production. In such a scenario, municipal authorities need to develop a strategy that would minimize the MSW management cost while increasing the longevity of landfill sites.

There has been a growing interest in converting waste to energy, a popular idea in South Asia [31]. Another way to reduce the use of plastics bags is to impose a ban and enforce the ban with significant fines. But without strict enforcement of the ban with sufficient fines, such a ban would fail miserably [18]. The ban would also increase the administrative costs of the cities as strict enforcement of the ban requires constant monitoring. This study discusses an alternative option, i.e., material recovery and recycling of plastic waste with additional tariffs on the import of plastic materials.

Recycling and reusing plastic would contribute to the following: reduce the volume of waste to be collected and disposed of in landfills while generating revenue. However, it requires an integrated approach involving all stakeholders–from members of households who segregate waste at source to collectors and recyclers. Usually, incentives motivate stakeholders to participate in environmental management programs [32,33]. In plastic waste management, a household could earn extra income from plastic waste if they segregate waste at the source. Similarly, waste pickers could also earn more once improved recycling activities are in place [34].

The results suggest that ISSWM not only collects, transfers and disposes of the waste but also minimizes the cost of MSW management by generating revenue from recovered waste material [26]. This could offset some portion of the MSW management cost, which is one of the more expensive activities of municipal authorities. This study suggests that revenue generated from recovered plastic could recover up to 20% of the MSW management cost. But the analysis excludes the indirect benefits of plastic recycling such as reduction in oil usage and carbon dioxide emissions as well as lowering the quantities of plastic waste dumped ensuring thereby the longevity of landfill sites [22]. In addition, recycling can also reduce the demand for virgin raw materials for the production of plastic items, reducing thereby the volume of plastic imports and the trade deficit. Accounting for these indirect benefits will increase the financial as well as the environmental contribution of recovered plastic waste in MSW management.

Even in conservative estimates that include only direct benefits, the generated revenue outweighs the plastic-related solid waste management cost in the given material recovery rate and collection efficiency. This corroborates the findings of another study according to which the financial benefits generated from properly managed construction waste offsets the management cost of the particular waste [21].

The results suggest that improving the material recovery rate would be more effective than increasing collection efficiency. However, both should be improved simultaneously to enhance the efficiency of MSW management. These inputs could be improved through appropriate infrastructure or policy instruments [35,36]. For instance, revenue could be increased through additional collection and recovery of high-value materials [35]. Improvement in the material recovery rate would require the introduction and enforcement of packaging standards and at-source waste segregation strategies [22,37]. Recovery and recycling of material from waste, however, is a complicated process. ISSWM requires several complementary policies and a supportive environment [7]. The experience of MSW management in Japanese municipalities suggests that the cost of MSW management depends on scale, segregation at household level, cooperation of adjacent municipalities in integrated management, and the manner in which the service is being provided [8]. Private sector engagement reduces cost and increases effectiveness [8,9]. An important aspect of increasing recovered material is segregation of waste at household level. It is hard to recover plastic from mixed waste as households pack mixed waste in plastic bags, making it almost impossible to segregate. In such a situation, promoting kitchen gardens or scaling up waste-to-energy-type biogas could consume the organic household waste so that easy-to-segregate waste enters the collection channel [10]. Enforcing recyclable packaging material could help circulate the same material and reduce the release of plastic waste into the environment [4]. However it would only work if segregation-at-source and recycling are practiced [11].

The quality of plastics and packaging materials could also be controlled by setting standards for those importing such materials with proper tax incentives. Additional import tariffs would have three benefits: i) it would immediately discourage the unnecessary use of plastics as plastic-related goods will become relatively expensive; ii) it would provide additional revenue to fill in the revenue gap in managing MSW properly; and iii) it would encourage promotion of substitutes for plastic in the medium to long term, which will help in achieving the goal of reducing plastic waste in the environment. According to the analysis in this paper, less than 1% of additional import duty on the import of plastic-related goods would generate sufficient revenue to bridge the resource gap in managing plastic waste if it were to coincide with the current collection efficiency and recovery rates of plastic waste.

Generally, a change in efficiency in collection and material recovery carries cost implications. However, changing the collection efficiency is difficult in the context of Nepal as municipalities are heterogeneous in terms of population density, waste production and accessibility. This heterogeneity compounds the difficulty of estimating the marginal cost of collection efficiency. For instance, some municipalities are characterized by sparsely populated suburban areas that may result in a high collection cost whereas some other municipalities would be able to improve the collection efficiency through predictable services [9]. Therefore, this study uses the average cost of per ton of MSW with a collection efficiency of up to 94%.

However, accounting for indirect effects of proper MSW management [38] as well as recovery of other waste materials such as paper and metals would assist in the recovery of such costs because segregation of plastic would also result in the segregation of other materials. It is also important to understand how the management cost changes with a change in the per unit marginal recovery rate and collection efficiency. While the expense associated with improving the material recovery rate through improved standards will be borne, in particular, by the consumer of the commodities, the expenditure incurred in improving collection efficiency will be shouldered by the waste management actors.

The financial component of the sustainable financing of MSW management is market-driven. Hence, the price of recovered materials is another determinant of revenue. This price may fluctuate due to volatile scrap market prices as requirements of scrap buyers could change over time [39]. If this were to happen, government interventions would be required to maintain the market price of scrap materials. Otherwise, households would be demotivated from segregating recyclable material at their homes and selling it to collectors; similarly, waste pickers may change occupations. Thus, one way to provide incentives for better recovery of plastic waste is to increase the import duty on plastic raw materials. In the short run, this would, in addition, generate revenue to finance plastic waste management. At the same time, it would encourage users to look for alternatives to plastics. The analysis suggests that less than 1% of import duty on the value of imported plastics materials would help finance the cost of managing plastic waste in Nepal.

## Conclusion

Sustainable financing hinges on both the financial and management components of waste management. Promoting a circular economy (i.e., reduce, reuse and recycle) would create opportunities for generating the much-needed resources for MSW management. The recovery of waste materials would produce several direct and indirect benefits. For instance, the estimated revenue generated from recovered plastic waste would come to 1.38 times the cost of managing plastic-related waste. In addition, it would prevent 4,220 tons of plastic waste from entering the environment annually, which would have additional environmental benefits. In low-performing cases where the collection cost is high and revenue is low, i.e., the revenue collected from recovered and recycled material is only 22% of the plastic-related waste management costs, which would require in turn additional resources, the cost could be met by imposing an additional 1% tax on the value of imported plastic-related material.

Nevertheless, sustainable MSW would require an integrated approach to designing and implementing management activities from source to landfill site that would link and engage all stakeholders. This study indicates that the financing of MSW management would depend on the effective management of municipal waste. Increased collection efficiency of waste material and material recovery rate would reduce the financial burden on municipal authorities. For example, collecting half of the MSW and recovering 15% of plastic material from the collected MSW would cover the cost of managing plastic-related waste. However, there is a possibility that the flow of financial resources could fluctuate based on recovery rate, collection efficiency, and price of recovered plastic. Thus, in the long term, additional policy interventions such as standardising of packaging materials and infrastructure development would help in managing MSW better.

This study only covers the financial and management aspects of ISSWM in Nepal. Future studies could focus on other aspects, particularly, stakeholder interests, policy coherence, and household behaviour in developing an ISSWM framework for Nepalese municipalities.

## Supporting information

S1 Data (DOCX)

## References

[1] DESA UN. Revision of world urbanization prospects. UN Department of Economic and Social Affairs; 2018.

[2] Minghua Z, Xiumin F, Rovetta A, Qichang H, Vicentini F, Bingkai L, et al Municipal solid waste management in Pudong New Area, China. Waste Manag. 2009;29: 1227–1233. 10.1016/j.wasman.2008.07.016 18951780

[3] Rowe RK, Armstrong MD, Cullimore DR. Mass loading and the rate of clogging due to municipal solid waste leachate. Can Geotech J. 2000;37: 355–370.

[4] Boldrin A, Andersen JK, Møller J, Christensen TH, Favoino E. Composting and compost utilization: Accounting of greenhouse gases and global warming contributions. Waste Manag Res. 2009;27: 800–812. 10.1177/0734242X09345275 19748950

[5] Figueroa VK, Mackie KR, Guarriello N, Cooper CD. A robust method for estimating landfill methane emissions. J Air Waste Manage Assoc. 2009;59: 925–935. 10.3155/1047-3289.59.8.925 19728486

[6] Pervin IA, Rahman SM, Nepal M, Hague AE, Karim H, Dhakal G. Adapting to urban flooding: A case of two cities in South Asia. Water Policy 2020;22(S1), 162–188.

[7] Visvizi A, Lytras MD, Damiani E, Mathkour H. Policy making for smart cities: Innovation and social inclusive economic growth for sustainability. J Sci Technol Policy Manag. 2018;9: 126–133.

[8] Medvedev A, Fedchenkov P, Zaslavsky A, Anagnostopoulos T, Khoruzhnikov S. Waste management as an IoT-enabled service in smart cities Internet of Things, Smart Spaces, and Next Generation Networks and Systems. Springer; 2015 pp. 104–115.

[9] Rai RK, Nepal M, Khadayat MS, Bhardwaj B. Improving municipal solid waste collection services in developing countries: A case of Bharatpur Metropolitan City, Nepal. Sustainability. 2019 10.3390/su11113010

[10] Rai RK, Bhattarai D, Neupane S. Designing solid waste collection strategy in small municipalities of developing countries using choice experiment. J Urban Manag. 2019 10.1016/j.jum.2018.12.008

[11] Hazra T, Goel S. Solid waste management in Kolkata, India: Practices and challenges. Waste Manag. 2009;29: 470–478. 10.1016/j.wasman.2008.01.023 18434129

[12] Sujauddin M, Huda SMS, Hoque ATMR. Household solid waste characteristics and management in Chittagong, Bangladesh. Waste Manag. 2008;28: 1688–1695. 10.1016/j.wasman.2007.06.013 17845843

[13] Henry RK, Yongsheng Z, Jun D. Municipal solid waste management challenges in developing countries–Kenyan case study. Waste Manag. 2006;26: 92–100. 10.1016/j.wasman.2005.03.007 16006111

[14] Guerrero LA, Maas G, Hogland W. Solid waste management challenges for cities in developing countries. Waste Manag. 2013;33: 220–232. 10.1016/j.wasman.2012.09.008 23098815

[15] Almazán-Casali S, Alfaro JF, Sikra S. Exploring household willingness to participate in solid waste collection services in Liberia. Habitat Int. 2019;84: 57–64.

[16] Ngoc UN, Schnitzer H. Sustainable solutions for solid waste management in Southeast Asian countries. Waste Manag. 2009;29: 1982–1995. 10.1016/j.wasman.2008.08.031 19285384

[17] Owusu G, Oteng-Ababio M, Afutu-Kotey RL. Conflicts and governance of landfills in a developing country city, Accra. Landsc Urban Plan. 2012;104: 105–113.

[18] Bhardwaj B, Baland J-M, Nepal M. What makes a ban on plastic bags effective? The case of Nepal. Environ Dev Econ. 2020; 25(2): 95–114.

[19] Lohri CR, Camenzind EJ, Zurbrügg C. Financial sustainability in municipal solid waste management–Costs and revenues in Bahir Dar, Ethiopia. Waste Manag. 2014;34: 542–552. 10.1016/j.wasman.2013.10.014 24246579

[20] Shekdar A V. Sustainable solid waste management: An integrated approach for Asian countries. Waste Manag. 2009;29: 1438–1448. 10.1016/j.wasman.2008.08.025 19081236

[21] Begum RA, Siwar C, Pereira JJ, Jaafar AH. A benefit–cost analysis on the economic feasibility of construction waste minimisation: The case of Malaysia. Resour Conserv Recycl. 2006;48: 86–98. 10.1016/j.resconrec.2006.01.004

[22] Hopewell J, Dvorak R, Kosior E. Plastics recycling: Challenges and opportunities. Philos Trans R Soc B Biol Sci. 2009;364: 2115–2126. 10.1098/rstb.2008.0311 PMC2873020 19528059

[23] Asian Development Bank. Solid waste management in Nepal: Current status and policy recommendations. Manila, The Philippines: Asian Development Bank (ADB); 2013.

[24] Pickin J, Randell P. Australian national waste report 2016. Dep Environ Energy. 2017.

[25] Khalisisi. What we buy? 2019 [cited 7 Jan 2019]. https://www.khaalisisi.com/#what-we-buy.

[26] Wilson DC, Velis CA, Rodic L. Integrated sustainable waste management in developing countries Proceedings of the Institution of Civil Engineers: Waste and Resource Management. Thomas Telford; 2013 pp. 52–68.

[27] Department of Customs [?]. Foreign Trade Statistics FY 2074/75 (2017/18). Monthly Foreign Trade Statistics based on First Month Data of Fiscal Year. Kathmandu; 2017.

[28] Phuyal N. Solid waste management baseline study in Ilam Municipality. Kathmandu: Unpublished Report, 2012.

[29] United Nations. World Urbanization Prospects: The 2018 Revision. New York; 2018.

[30] Yukalang N, Clarke B, Ross K. Solid waste management solutions for a rapidly urbanizing area in Thailand: Recommendations based on stakeholder input. Int J Environ Res Public Health. 2018;15: 1302. 10.3390/ijerph15071302 PMC6068799 29933621

[31] Haque AKE, Lohano HD, Mukhopadhyay P, Nepal M, Shafeeqa F, Vidanage SP. NDC pledges of South Asia: Are the stakeholders onboard? Clim Change. 2019;155(2): 237–244.

[32] Reschovsky JD, Stone SE. Market incentives to encourage household waste recycling: Paying for what you throw away. J policy Anal Manag. 1994;13: 120–139.

[33] Rai RK, Nepal M, Bhatta LD, Das S, Khadayat MS, Somanathan E, et al Ensuring water availability to water users through incentive payment for ecosystem services scheme: A case study in a small hilly town of Nepal. Water Econ Policy. 2017; 1850002. 10.1142/S2382624X18500029

[34] McBean EA, Del Rosso E, Rovers FA. Improvements in financing for sustainability in solid waste management. Resour Conserv Recycl. 2005;43: 391–401.

[35] Ip K, Testa M, Raymond A, Graves SC, Gutowski T. Performance evaluation of material separation in a material recovery facility using a network flow model. Resour Conserv Recycl. 2018;131: 192–205.

[36] Gu F, Ma B, Guo J, Summers PA, Hall P. Internet of things and Big Data as potential solutions to the problems in waste electrical and electronic equipment management: An exploratory study. Waste Manag. 2017;68: 434–448. 10.1016/j.wasman.2017.07.037 28757222

[37] Matter A, Dietschi M, Zurbrügg C. Improving the informal recycling sector through segregation of waste in the household–The case of Dhaka Bangladesh. Habitat Int. 2013;38: 150–156.

[38] Nepal M, Rai RK, Khadayat MS, Somanathan E. Value of cleaner neighborhoods: Application of hedonic price model in low income context. World Development. 2020;131: 104965.

[39] Ragaert K, Delva L, Van Geem K. Mechanical and chemical recycling of solid plastic waste. Waste Manag. 2017;69: 24–58. 10.1016/j.wasman.2017.07.044 28823699

