# Supplementary material for: Sustainable financing for municipal solid waste management in Nepal
Source: PLoS One. 2020 Aug 20;15(8):e0231933. doi: 10.1371/journal.pone.0231933 (PMC7440930; doi:10.1371/journal.pone.0231933)
Supplement: S1 Data — (DOCX) [file pone.0231933.s001.docx]

**Survey Questionnaire**

This study intends to analyze the cost of plastic waste management and ways it can be financed. This questionnaire interview will be administered to the selected respondent among producer, importer, government officials, local level representative/officials and other concerned stakeholders only after obtaining a verbal consent from the respondent to participate in the survey. This interview will take approximately 30 minutes. The respondent will not receive any direct benefit for participating in this study. But the findings of this study will be useful for policy formulation for plastic waste management policy. There might be some risks as the respondent will have to expose some household information, but this information will be kept strictly confidential and will be used for comprehensive analytical purpose only. I would like to request you for your cooperation by participating in this survey. If you agree to participate, we shall proceed. If you do not agree, you may refuse to participate in the survey.

Do you agree to participate in this survey? ………… Yes …………………….No

**Part A: Common Questions for All Groups**

**SECTION A: IDENTIFICATION**

| cs1 | cs2 | cs3 | cs4 |
| --- | --- | --- | --- |
| Category | Contact person | Gender | Education |
| 1. Importer 2. Producer 3. Recycler 4. Collector center 5. Collector | Name | 1. Male 2. Female | No of class year |
|  | Mobile: |  |  |

**SECTION B: COMMON QUESTION**

1. **At first I would like to present some statement about plastic and the way it might pollute environment. Please respond.:**

| SN | Do you…. | **Response** | | | | | Don’t know |
| --- | --- | --- | --- | --- | --- | --- | --- |
|  |  | Strongly Agree | Agree | Neutral | Disagree | Strongly disagree |  |
| 1 | Plastic is a pollutant. |  |  |  |  |  |  |
| 2 | Plastic has now become a major source of environmental pollution. |  |  |  |  |  |  |
| 3 | Most of the plastic is imported |  |  |  |  |  |  |
| 4 | Most of the plastic pollutant are comes with packaging and carry bag. |  |  |  |  |  |  |
| 5 | Use of plastic goods is increasing? |  |  |  |  |  |  |
| 6 | Despite imported, Plastic waste management is local level responsibility. |  |  |  |  |  |  |
| 7 | Plastic waste management is costly; thus, the local government should be funded for its management |  |  |  |  |  |  |
| 9 | To get the fund plastic good must be charged for pollution. |  |  |  |  |  |  |
| 10 | Do you think fund is a problem in SWM at local level |  |  |  |  |  |  |

11 Who should be responsible for pollution from plastic goods; Please prioritize

| Responsible agent: | Priority |
| --- | --- |
| 1. Producer |  |
| 1. Consumer |  |
| 1. Local government |  |
| 1. Central government |  |
| 1. Other if any |  |

12. Is it justifiable for municipality to invest on plastic waste which is majorly transported at local level as constituent of traded goods? Yes ……………………… no ……………………..

**13. Who should manage?**

| SN | Cost factor | Whose Duty to manage | | | |
| --- | --- | --- | --- | --- | --- |
|  |  | Household | Municipality | Central Government | Producer |
| 1 | Segregation |  |  |  |  |
| 2 | Collection |  |  |  |  |
| 3 | Transportation |  |  |  |  |
| 4 | Landfill management |  |  |  |  |
| 5 | Other |  |  |  |  |

**14. Who should cover the cost of plastic waste management?**

| Cost factor | Who should pay for the plastic waste? | | | |
| --- | --- | --- | --- | --- |
|  | User (Household) | Manager (Municipality) | Tax collector (Central Government) | Producer (Importer) |
| Segregation |  |  |  |  |
| Collection |  |  |  |  |
| Transportation |  |  |  |  |
| Landfill management |  |  |  |  |

**15.** Plastic goods are charged around 30% of import value these days. Based on harm it does, is it:

i. less than the harm it does to environment

ii. higher than the environmental damage it does

iii. Fair %

**16. Charging plastic goods during import:**

To meet the cost of plastic waste management -at current practice at local level, central government has to top-up 5% tax on total value of import for plastic waste management. Do you support ;

1. charging additional 5% on plastic goods? A) yes b) no c) don’t know
2. And transfer it to local level for plastic waste management? A) yes b) no c) don’t know

17. Are you in-favor of increasing cost of plastic bags (by increasing charge which could be any 5%^[[1]](#footnote-1)^) to make bio-degradable plastic slightly cheaper than plastic goods?

Yes …………………. No ……………

18. if the price of the plastic goods (say bag and cups) is equal to bio-degradable goods will start using them?

A. Yes B. No

19 The cost of plastic bag or Plastic cup will become … times costly (say from NRs 5 to NRs …) to make bio-degradable bags and Cups competitive to biodegradable then will you accept it?

A. Yes B. No

20. Understanding the attitude toward Supply side: Here are some statement. Please respond accordingly.

| SN | Do you: | Response | | | | | Don’t know |
| --- | --- | --- | --- | --- | --- | --- | --- |
|  |  | Strongly Agree | Agree | Neutral | Disagree | Strongly disagree |  |
| 1 | Increasing plastic tax/price will increase the price of plastic? |  |  |  |  |  |  |
| 2 | Increase in price of plastic will increase the price of recyclable plastic? |  |  |  |  |  |  |
| 3 | Increase in price of recyclable plastic will increase collection of recyclable plastic? |  |  |  |  |  |  |
| 4 | Increase in recyclable plastic collection will reduce the import of plastic bag? |  |  |  |  |  |  |

21. User behavioral change: Here, I have some statement about the use behavior of plastic bag. Please respond from strongly agree to strongly disagree.

| SN | Do you: | Response | | | | | Don’t know |
| --- | --- | --- | --- | --- | --- | --- | --- |
|  |  | Strongly Agree | Agree | Neutral | Disagree | Strongly disagree |  |
| 1 | Increasing plastic tax/price will increase the price of plastic? |  |  |  |  |  |  |
| 2 | Increase in price of plastic will reduce the demand of plastic material |  |  |  |  |  |  |
| 3 | Increased price will encourage reuse of plastic goods? |  |  |  |  |  |  |
| 4 | Increase in price of plastic will encourage bio-degradable product in the market. |  |  |  |  |  |  |

22. Which Scenario do you want to choose?

| SN | Level | Priority |
| --- | --- | --- |
| 1 | Continue current practice (increasing use of plastic and pollution increasing) |  |
| 2 | Top-up plastic bag charge/tax during import (5%) and transfer it with condition that plastic waste will be managed with in standard as per funding guideline. |  |
| 3 | Increase the cost of plastic that equals the cost of bio-degradable plastics |  |
| 4 | Use plastic levy to reduce use of plastic bag and fund municipalities to get better plastic SWM with segregation and selling of plastic to match central transfer |  |

**Part B: Questionnaire specific to particular groups**

1. **Questionnaire for Collectors**

| 1 | 2 | 3 | 4 |
| --- | --- | --- | --- |
| Total recycling material collected per year | How much you **pay** when you sell these recyclable plastic good NRs per KG | How much you used to **get** when you sell these recyclable plastic good during 2012  NRs per KG | How much you **get** when you sell these recyclable plastic good NRs per KG |
| KG | Plastic bag: …………..  Plastic bottle: ………….  Utensils : ………….  Others: ………. | Plastic bag: …………..  Plastic bottle: ………….  Utensils : ………….  Others: ………. | Plastic bag: …………..  Plastic bottle: ………….  Utensils : ………….  Others: ………. |
|  |  |  |  |

| 5 | 6 | 7 | 8 |
| --- | --- | --- | --- |
| What is the demand for recycled plastic? | Can you rank the goods based on easy recycling? | What percentage of total plastic used is recycled plastic? | What is the major problem |
| 1. Very high 2. High 3. Low | Plastic bag: …………..  Plastic bottle: ………….  Utensils : ………….  Others: ………. | Plastic bag: …………..  Plastic bottle: ………….  Utensils : ………….  Others: ………. | 1. Low price 2. Getting material 3. Getting market 4. Management |

9. Can you list the item that is not recycled or collected to sell?

1. ________________________
2. ________________________
3. ___________________________
4. ____________________________
5. __________________

10 What are the major problem in recyclable waste collection?

11 What could facilitate more plastic goods be collected for recycling?

1. ______________________________
2. _____________________________
3. ________________________________

12. Any other issues.

1. **Questionnaire for Local Government Officials**

| 1 | 2 | 3 | 4 |
| --- | --- | --- | --- |
| Can you guess what % if municipal budget is spend in SWM? | Why doesn’t plastic have pollution charge? | Are you in favor of making plastic as costly as Bio-degradable? | Plastic good pays on an average 30-54% tax, do you think this can be increased? |
| NRs | 1. Private 2. Municipality 3. other | 1. Yes 2. No | 1. Yes 2. No |
|  |  |  |  |

| 5 | 6 | 7 |
| --- | --- | --- |
| Is it good to add charge plastic good to manage its SWM | Do you believe that funding plastic SW will increase its management quality? | Do you think it is possible to charge plastic at import and transfer it to local government for SW management? |
| 1. Yes 2. No | 1. Yes 2. No | 1. Yes 2. No |
|  |  |  |

8 What could be the implication of plastic charge change?

9 Do you think it is possible to increase the plastic bag tax so that bio-degradable plastic gets space for competition?

10 Any alternative ideas that government could prefer for plastic use reduction?

1. **Questionnaire for NGOs**

| 1 | 2 | 3 | 4 |
| --- | --- | --- | --- |
| Plastic face 30% tax, do you think this is sufficient? | Is it good to impose pollution charge to on plastic good? | Are you in favor of making plastic as costly as Bio-degradable? | What do you support? Ban or Pollution charge? |
| 1. high 2. Sufficient 3. low | 1.V good 2 Good  3 normal 4. Bad  5 very bad. 6 don’t know | 1. Yes 2. No | 1. Ban 2. Pollution charge |
|  |  |  |  |

| 5 | 6 | 7 |
| --- | --- | --- |
| Is it good to add charge plastic good to manage its SWM | Do you believe that funding plastic SW at local level will increase its management quality? | Do you think it is possible to charge plastic at import and transfer it to local government for SW management? |
| 1. Yes 2. No | 1. Yes 2. No | 1. Yes 2. No |
|  |  |  |

8 What could be the implication of plastic charge change?

9 Do you think it is possible to increase the plastic bag tax so that bio-degradable plastic gets space for competition?

1. Any alternative ideas that government could prefer for plastic use reduction?

**4. Questionnaire for Policy Makers**

| 23 | 24 | 25 | 26 |
| --- | --- | --- | --- |
| Can you guess what % if municipal budget is spend in SWM? | Why doesn’t plastic have pollution charge? | Are you in favor of making plastic as costly as Bio-degradable? | Plastic good pays on an average 30-54% tax, do you think this can be increased? |
| NRs | 1. Private 2. Municipality 3. other | 1. Yes 2. No | 1. Yes 2. No |
|  |  |  |  |

| 27 | 28 | 29 |
| --- | --- | --- |
| Is it good to add charge plastic good to manage its SWM | Do you believe that funding plastic SW will increase its management quality? | Do you think it is possible to charge plastic at import and transfer it to local government for SW management? |
| 1. Yes 2. No | 1. Yes 2. No | 1. Yes 2. No |
|  |  |  |

What could be the implication of plastic charge change?

Do you think it is possible to increase the plastic bag tax so that bio-degradable plastic gets space for competition?

Any alternative ideas that government could prefer for plastic use reduction?

1. From author analysis [↑](#footnote-ref-1)
